# Sortilin Modulates Schwann Cell Signaling and Remak Bundle Regeneration Following Nerve Injury

**DOI:** 10.3389/fncel.2022.856734

**Published:** 2022-05-11

**Authors:** Maj Ulrichsen, Nádia P. Gonçalves, Simin Mohseni, Simone Hjæresen, Thomas L. Lisle, Simon Molgaard, Niels K. Madsen, Olav M. Andersen, Åsa F. Svenningsen, Simon Glerup, Anders Nykjær, Christian B. Vægter

**Affiliations:** ^1^Danish Research Institute of Translational Neuroscience – DANDRITE, Nordic EMBL Partnership for Molecular Medicine, Department of Biomedicine, Aarhus University, Aarhus, Denmark; ^2^Department of Biomedical and Clinical Sciences, Linköping University, Linköping, Sweden; ^3^Neurobiological Research, Faculty of Health Sciences, Department of Molecular Medicine, University of Southern Denmark, Odense, Denmark; ^4^Department of Biomedicine, Aarhus University, Aarhus, Denmark; ^5^Department of Neurosurgery, Aarhus University Hospital, Aarhus, Denmark; ^6^Center of Excellence PROMEMO, Aarhus University, Aarhus, Denmark

**Keywords:** nerve injury, NT-3, TrkC, sortilin, Schwann cell, Remak bundle

## Abstract

Peripheral nerve regeneration relies on the ability of Schwann cells to support the regrowth of damaged axons. Schwann cells re-differentiate when reestablishing contact with the sprouting axons, with large fibers becoming remyelinated and small nociceptive fibers ensheathed and collected into Remak bundles. We have previously described how the receptor sortilin facilitates neurotrophin signaling in peripheral neurons *via* regulated trafficking of Trk receptors. This study aims to characterize the effects of sortilin deletion on nerve regeneration following sciatic crush injury. We found that *Sort1*^–^*^/^*^–^ mice displayed functional motor recovery like that of WT mice, with no detectable differences in relation to nerve conduction velocities and morphological aspects of myelinated fibers. In contrast, we found abnormal ensheathment of regenerated C-fibers in injured *Sort1*^–^*^/^*^–^ mice, demonstrating a role of sortilin for Remak bundle formation following injury. Further studies on Schwann cell signaling pathways showed a significant reduction of MAPK/ERK, RSK, and CREB phosphorylation in *Sort1*^–^*^/^*^–^ Schwann cells after stimulation with neurotrophin-3 (NT-3), while Schwann cell migration and myelination remained unaffected. In conclusion, our results demonstrate that loss of sortilin blunts NT-3 signaling in Schwann cells which might contribute to the impaired Remak bundle regeneration after sciatic nerve injury.

## Highlights

- Sortilin ablation results in Remak bundle pathology after nerve injury.
- *In vitro* studies demonstrate blunted NT-3 signaling in sortilin-deficient Schwann cells.

## Introduction

Schwann cells are the axon-ensheathing glial cells of the peripheral nervous system (PNS), generally divided into myelinating and non-myelinating subtypes, both being essential for maintaining normal nerve functions. The myelinating Schwann cells form a multi-layered myelin sheath by spirally wrapping plasma membrane around a segment of a single caliber axon. In contrast, the non-myelinating Schwann cells surround and segregate groups of several small-diameter axons into Remak bundles (Jessen and Mirsky, 2002).

Following peripheral nerve injury, the process of Wallerian degeneration sets the stage for subsequent regeneration. This process is characterized by the morphological and molecular changes that occur in the distal part of an injured peripheral nerve, where the damaged axon and myelin debris become degraded. The Schwann cells dedifferentiate into their repair phenotype, and the macrophages infiltrate the injured nerve to partake in myelin degradation (Gaudet et al., 2011). A population of repair Schwann cells proliferate and migrate, forming the characteristic bands of Büngner to provide guidance and growth factor support (including secreted neurotrophins) for the regenerating axons, stimulate ensheathing of small-caliber fibers to reform Remak bundles, and facilitate remyelination of large-caliber axons (Gaudet et al., 2011; Jessen and Arthur-Farraj, 2019; Jessen and Mirsky, 2019, 2022). Neurotrophins elicit trophic signaling for sprouting axons but are also reported to regulate Schwann cell functions. Thus, *in vitro* studies have shown how neurotrophin-3 (NT-3) and brain-derived neurotrophic factor (BDNF) appear to have opposing effects on Schwann cell migration and myelin formation, with NT-3/TrkC enhancing Schwann cell migration but inhibiting myelin formation (Yamauchi et al., 2003, 2004, 2005b), whereas BDNF/p75^NTR^ inhibits Schwann cell migration and enhance myelin formation (Cosgaya et al., 2002; Tolwani et al., 2004; Yamauchi et al., 2004).

Intracellular signaling pathways are activated upon binding of neurotrophins to their cognate Trk receptors, resulting in increased phosphorylation of downstream messengers such as protein kinase B (Akt) as well as the mitogen-activated protein kinase (MAPK/ERK). Transcriptional events are induced by MAPK/ERK *via* members of the ribosomal s6 kinase (RSK) family and CREB transcription, ultimately modulating the cell cycle, neurite outgrowth, and synaptic plasticity (Chao, 2003). The activation of MAPK/ERK can be observed early after injury (Sheu et al., 2000) and is implicated in the injury-induced dedifferentiation of Schwann cells (Harrisingh et al., 2004). Accordingly, MAPK/ERK activation in Schwann cells is sufficient to drive the dedifferentiation of myelinating Schwann cells to a progenitor-like state in peripheral adult nerves, causing severe loss of motor function with rapid remyelination and neurological recovery occurring after inhibition of the MAPK/ERK activation (Napoli et al., 2012). Of further support has been how sustained MAPK/ERK activation in Schwann cells resulted in delayed functional recovery and morphological defects in both myelinated and non-myelinated fibers after injury (Cervellini et al., 2017).

Sortilin is a member of the Vps10p sorting receptor family and is involved in the transport of a wide variety of intracellular proteins between several cellular compartments (Willnow et al., 2008; Nykjaer and Willnow, 2012). We have previously described how the sortilin receptor, abundantly expressed in the central nervous system and the PNS, facilitates the phosphorylation of MAPK/ERK in primary sensory neurons by associating with Trk receptors to mediate their subcellular trafficking (Vaegter et al., 2011). As Schwann cells express sortilin (Gonçalves et al., 2020) as well as TrkC receptors, we asked if sortilin is similarly involved in neurotrophin signaling in these cells, and more specifically if ablation of sortilin *in vivo* would affect the morphological and/or functional aspects of sciatic nerve regeneration. We have found that the lack of sortilin elicits a blunted NT-3 signaling in cultured Schwann cells, which might contribute to the impaired Remak Schwann cell regeneration following nerve injury.

## Materials and Methods

### Primary Rat Schwann Cell Cultures

Primary Schwann cell cultures were prepared from neonatal rat sciatic nerves according to Kim and Maurel (2009) with few alterations. Pregnant wild-type (WT) Sprague–Dawley rats were obtained from Janvier Labs (France). *Sort1*^–^*^/^*^–^ Sprague–Dawley rats were bred at Aarhus University (Gonçalves et al., 2020). In brief, sciatic nerves were dissected from P1-P3 Sprague–Dawley rat pups and placed in ice-cold Leibovitz’s L-15 medium (Gibco, Cat. #11415). Following dissection, the harvested nerves were digested with 0.25% trypsin (Gibco, Cat. #25200-072) and 0.1% collagenase I (Sigma, Cat. #C9722) for 30 min at 37°C and dissociated by trituration in DMEM (Sigma, Cat. #D0819) containing 10% FBS (Thermo Fisher, Cat. #10270) before being plated on poly-L lysine-coated culture dishes in DMEM supplemented with 10% FBS and Primocin™ (InvivoGen, Cat. #ant-pm-2). Contaminating fibroblasts were removed by 48 h incubation with the anti-metabolic agent Cytosine-ß-arabino furanoside hydrochloride (Ara-C; 10 μM final concentration, Sigma, Cat. #C1768), which kills mitotic cells. The cultures were washed twice with HBSS (Gibco, Cat. #14170-088) and the Schwann cells expanded in the growth medium [DMEM containing 10% FBS, recombinant human neuregulin-1-β1 EGF domain (10 ng/ml, R&D Systems, Cat. #396-HB-050) and forskolin (2.5 μM, Sigma, Cat. #F-6886)].

### Mouse Model and Surgical Procedures

Sciatic nerve crush injury was performed in both 12-weeks-old WT mice (Janvier Labs, France) and *Sort1*^–^*^/^*^–^ mice (Jansen et al., 2007). Mixed groups with both males and females were used throughout the experiments.

The animals were anesthetized with isoflurane (IsoFlo vet, Abbot). Eye ointment was applied to protect the eyes from drying during the procedure and a subcutaneous injection of buprenorphine (0.3 mg/ml; Temgesic, RB Pharmaceuticals, Cat. #521634) and ampicillin (0.25 mg/ml; Ampicillin PCD, PharmaCoDane, Cat. #175689) was administered before surgery, to minimize post-surgery pain and risk of infection. The thigh and legs were shaved and disinfected before application of lidocaine (10 mg/ml; AstraZeneca, Cat. #PS02671) and exposure of the sciatic nerve at the mid-thigh level by separation of the biceps femoris and the gluteus superficialis. The left sciatic nerve was crushed with a non-serrated clamp for 30 s (Varejo et al., 2004; Ronchi et al., 2010). A sham surgery was performed on the contralateral side, where the sciatic nerve was exposed and the skin sutured immediately after. Care was taken to minimize damage to the musculature to guarantee ideal conditions for functional recovery. Post-surgery analgesic (0.006 mg/ml buprenorphine) was supplied in the drinking water for a minimum of 2 days. The mice were housed with littermates and cared for in a pathogen-free environment, in a 12 h light/dark cycle and with water and chow *ad libitum*. All experiments were approved by the Danish Animal Experiments Inspectorate under the Ministry of Justice (permission no. 2017-15-0201-01192) and carried out according to the European Council directive, and institutional and national guidelines.

### Motor Analysis

Walking tract analysis (Mendes et al., 2015) was performed to access locomotor functional recovery before (0) and on days 3, 7, 9, 11, 18, 25, and 60 after injury (*n* = 6 mice per genotype). The mice were allowed to walk through a homemade catwalk consisting of a 50 cm × 5.5 cm corridor with a transparent Plexiglas floor to a box on the side of the catwalk containing bedding from their cages. Green light was sent horizontally through the Plexiglas floor and ambient red light was used to enhance contrast to the green light reflected down when the mouse placed a paw on the floor. The paw placement during walking was accurately determined from a video recorded with a camera below. A liner was placed on top of the Plexiglas floor. This way, the paw prints and gait could be analyzed in ImageJ by setting the scale accordingly to obtain print measurements.

The obtained footprints were then measured in ImageJ to calculate the sciatic functional index (SFI) using the empirical equation adapted for mice by Inserra et al. (1998): SFI = 118.9 × [(ETS-CTS)/CTS] − 51.2 × [(EPL-CPL)/CPL] − 7.5, where ETS represents operated experimental toe spread (distance between the first and fifth toes), CTS stands for control toe spread, EPL for operated experimental print length, and CPL for control print length. Footmarks made at the beginning of the trial were excluded and three analyzable walks were evaluated from each run for individual step parameter calculation. The pre-injured SFI values (time point = 0) were used as control for comparison. The SFI scores that we processed ranged from 0 to −130, with 0 representing normal or completely recovered nerve function and −100 or more, a non-functional nerve; thus, the mice who dragged their paws were arbitrarily assigned a value of −100.

The stride length and the step length (from the center of the right paws to the center of the left paws and vice versa) were measured in ImageJ from 3 consecutive steps. The step width and the angle of the paw to the mouse midline were additionally measured from the 3 consecutive steps. The mice who dragged their paws were arbitrarily assigned a value of 90°.

A grip strength test was performed after the walking tract analysis to evaluate the hind limb muscle strength as an indicator of motor regeneration and neuromuscular function before (0) and on days 3, 9, 11, 18, 25, 31, 38, 45, and 60 after injury. Combined hind limb grip strength was measured as tension force using the commercial grip strength meter from Bioseb (Chaville, France), which digitally displays the maximum force applied as the peak tension (in Newton) when the grasp is released. The mice were subjected to 5 consecutive trials and the maximum value was taken as an index of the hind limb strength.

### Nerve Conduction Velocities

Motor (sciatic) and sensory (sural) nerve conduction velocities (MNCV, SNCV, respectively) were performed 60 days after crush injury according to Oh et al. (2010) using a Viking Quest apparatus (Natus Neurology Incorporated, United States) (*n* = 6 mice per group). NCV was measured from the crush-injured side and from the contralateral side, which was subjected to sham surgery. Briefly, for the sural nerve, recording electrodes were placed in the dorsal part of the foot, with supramaximal stimulation at the ankle. Sural sensory NCV (m/s) was calculated by dividing the distance between the recording and stimulating electrodes (mm) by the onset latency (ms) of the sensory nerve action potential after supramaximal antidromic stimulation. Sciatic-tibial motor NCV was recorded by placing electrodes dorsally in the foot and orthodromically stimulating first at the ankle, then at the sciatic notch. The distance between the two sites of stimulation (mm) was then divided by the difference between the two onset latencies (ankle distance and notch distance, ms) to calculate the final sciatic-tibial motor NCV (m/s).

### Sciatic Nerve Histology and Axon Density Analysis

The operated mice (*n* = 6 per genotype) were euthanized 25 days after injury with intraperitoneal injections of pentobarbital (5 mg/ml). The mice were then transcardially perfused with ice-cold Tyrodes solution followed by fixation in ice-cold 5% glutaraldehyde in 300 mOsm phosphate buffer containing 0.1 M sucrose. The samples were postfixed in 1% osmium tetroxide for 1 h at 4°C and washed in the phosphate buffer. The samples were dehydrated in 50% ethanol and stained with 2% uranyl acetate in 50% ethanol. The samples were then dehydrated in a series of ascending concentrations of ethanol and 100% acetone. A three-step infiltration in a mixture of acetone-embedding medium (1:3, 1:1, 3:1) was performed before embedding (48 h at 60°C) in the Epoxy Embedding Medium Kit (Sigma-Aldrich, Sweden AB). The blocks were initially trimmed and sectioned using a Leica UC7 ultra microtome (Leica Microsystems GmbH, Vienna, Austria). Ultrathin sections (60 nm thickness) were collected onto formvar-coated copper slot grids, and counter-stained with uranyl acetate and lead citrate. Images were taken using a 100 kV transmission electron microscope (TEM, JEM 1230, JEOL Ltd., Tokyo, Japan). For each nerve, a panorama picture was created (x1,200) for orientation and quantification of myelinated fibers. For C-fiber quantification, 30 pictures were randomly taken (each third window; x15,000), and fibers were counted and normalized against the area to obtain fiber density.

### Immunohistochemistry

Sciatic nerves dissected 5 days after injury were fixed overnight with 4% PFA and cryoprotected in 25% (w/v) sucrose in DPBS. The tissue was embedded in OCT compound mounting medium (VWR, Cat. #361603E) and snap-frozen on dry ice using isopentane before sectioning with a Leica CM1900 cryostat. About 10 μm longitudinal sections were thawed at RT for 10 min and antigen retrieval was performed with Target Retrieval Solution (Dako, Cat. #S1699) for 30 min at 80°C. The sections were rinsed once in TBS (50 mM tris base and 150 mM NaCl, pH 7.4) and incubated in TBS containing 5% donkey serum and 0.3% Triton-X-100 to block the unspecific binding of antibodies. The sections were incubated with primary antibodies (Table 1) diluted in TBS containing 5% donkey serum overnight in a humidified chamber at 4°C. The following day, the sections were incubated for 4 h with secondary antibodies (Table 1) diluted in TBS containing 5% donkey serum and Hoechst 33258 was used for nuclear stain. The slides were mounted with Dako fluorescence mounting medium (Dako, Cat. #S3023), sealed with nail polish, and stored at 4°C.

**TABLE 1:** List of antibodies used.

Antibodies	Supplier, cat. no.	RRID	Dilution	Use
Mouse anti-βIII Tubulin	Promega, G7121	AB_430874	1:400	IF
Rabbit anti-Ki-67	Thermo Scientific, MA5-14520	AB_10979488	1:200	IF
Rabbit anti-S100β	Dako, Z0311	AB_10013383	1:400	IF/WB
Goat anti-sortilin	R&D Systems, AF2934	AB_2192424	1:100	IF
Donkey anti-rabbit IgG, Alexa Fluor 488	Invitrogen, A21206	AB_2535792	1:300	IF
Donkey anti-goat IgG, Alexa Fluor 488	Invitrogen, A11055	AB_2534102	1:300	IF
Donkey anti-sheep IgG, Alexa Fluor 488	Invitrogen, A11015	AB_141362	1:300	IF
Donkey anti-mouse IgG, Alexa Fluor 568	Invitrogen, A10037	AB_2534013	1:300	IF
Donkey anti-rabbit IgG, Alexa Fluor 647	Invitrogen, A31573	AB_2536183	1:300	IF
Mouse anti-β-Actin	Sigma, Cat. A5441	RRID:AB_476744	1:5,000	WB
Mouse anti-NTR3	BD Bioscience, 612100	AB_399471	1:1,000	WB
Rabbit anti-phospho-Akt (Ser473)	Cell signaling, 4060	AB_2315049	1:1,000	WB
Rabbit anti-phospho-p44/42 MAPK/ERK	Cell Signaling, 9101	AB_331646	1:1,000	WB
Rabbit anti-pRSK	Abcam, AB32413	AB_2181172	1:1,000	WB
Rabbit anti-pCREB	Cell Signaling, 9198	AB_2561044	1:1,000	WB
Rabbit anti-p75NTR	Abcam, 10494	AB_297233	1:1,000	WB
HRP swine anti-rabbit	Dako, P0217	AB_2728719	1:2,000	WB
HRP rabbit anti-mouse	Dako, P0260	AB_2636929	1:2,000	WB
HRP rabbit anti-goat	Dako, P0160	AB_2617143	1:200	WB
HRP rabbit anti-sheep	Dako, P0163		1:2,000	WB

Images were acquired with a Zeiss Axio Imager 2 microscope (Carl Zeiss, Germany) equipped with a Hamamatsu digital camera (ORCA-flash4.0 digital camera, model C11440-22CU, Hamamatsu Photonics Deutschland GmbH, Germany) and a Plan-Apochromat 20x/0.8 lens or the LSM 780 confocal microscope (Carl Zeiss) equipped with a 63x water-immersion objective or a 20x/0.8 M27 objective (Carl Zeiss). Subsequent image analysis was performed with ImageJ.

### Immunocytochemistry

The Schwann cells were fixed for 10 min in 4% formaldehyde and permeabilized with DPBS containing 0.1% Triton-X-100 (PanReac Applichem, Cat. #A4975) 3 times for 10 min. Unspecific binding of antibodies was blocked by subsequent incubation in DPBS containing 5% donkey serum and 1% BSA (Sigma, Cat. #A8806). The cells were then incubated with primary antibodies (Table 1) diluted in DPBS and containing 1% BSA overnight at 4°C. The samples were left for 1 h at RT before being washed 3 times for 10 min in DPBS and then incubated for 4 h with the secondary antibodies (Table 1), diluted in 1% BSA. Hoechst 33258 was used for nuclear staining (1:10.000, Sigma). The sections were then mounted with Dako Fluorescent mounting medium (Dako, Cat. #S3023) and sealed with nail polish.

### Western Blot Analysis

*Sort1*^–^*^/^*^–^ and WT primary Schwann cells were lysed in ice-cold lysis buffer [10 mM Tris–HCl and 1 mM disodium EDTA, pH 8.0 (Sigma-Aldrich, Cat. #93283)] containing 1% IGEPAL (Sigma-Aldrich, Cat. #I8896), cOmplete Mini (Roche, Cat. # 4719956001), and PhosStop (Roche, 4906845001), incubated for 30 min on ice and frozen overnight at −20°C.

Naïve mice (day 0) or crush-injured mice were euthanized by decapitation after isoflurane gas-induced anesthesia at days 1, 7, or 25 post-injury (*n* = 3 animals per time point/group). The sciatic nerves were lysed in TNE buffer supplemented with protease (cOmplete Mini, Roche) and phosphatase inhibitors (PhosStop, Roche), after which a 10.000 *g* centrifugation for 10 min at 4°C was performed. The supernatant was stored at −20°C until use. A vacuum concentrator was used to increase protein concentration in the lysates, when necessary. Total protein concentration for both cell and tissue lysates was determined with the Bicinchoninic acid assay (Sigma, Cat. #BCA1-1KT). About 50 or 100 μg total protein was mixed with NuPAGE LDS sample buffer (Life Technologies, Cat. #NP0008) and a minimum of 10 mM dithioerythritol (DTE, AppliChem). The protein samples were separated on 4–12% Bis-Tris protein gels (Life technologies) in MOPS SDS running buffer (Thermo Fisher, Cat. #NP0001) containing NuPAGE Antioxidant (Invitrogen, Cat. #NP0005) and electroblotted with nitrocellulose iBlot Gel Transfer Stacks (Invitrogen, Cat. #IB301001) using the iBlot DryBlotting System (Invitrogen) according to manufactures guidelines. The membranes were subsequently blocked in TST buffer [50 mM Tris base, 500 mM NaCl, and 0.05% Tween-20 (PanReac Applichem, Cat. #A4974)] containing 2% skimmed milk powder and 2% Tween-20 and subsequently incubated with primary antibodies (Table 1), diluted in blocking buffer overnight at 4°C. The following day, the membranes were washed 3 times for 5 min in washing buffer (2 mM CaCl2, 1 mM MgCl2, 10 mM Hepes, and 140 mM NaCl) containing 0.2% skimmed milk powder and 0.05% Tween-20 and incubated for 1 h at RT with HRP conjugated secondary antibodies (Table 1). The membranes were then washed 3 times for 5 min and developed with Amersham ECL Prime Western Blotting Detection Reagent (GE Healthcare, Cat. #RPN2236). Fuji film LAS4000 was used for visualization and densitometry.

### Viability Assay and Ki-67 Proliferation Assay

The viability of Schwann cells was assessed by seeding Schwann cells on poly-L lysine-coated coverslips (250.000 cells/ml) in DMEM containing 10% FBS, at 37°C and 5% CO_2_. The following day, the medium was replaced with or without NT-3 (50 ng/ml, Sigma, Cat. #SRP3128). The viability assay was performed 24 h after stimulation, according to the manufacture’s protocol (Live/Death Viability/Cytotoxicity Kit, Molecular Probes, Cat. #L3224). Calcein-positive (live) cells and ethidium homodimer-1 (dead) cells were counted and the percentage of live cells was calculated (*n* = 4 for each genotype).

The proliferation rate was assessed (*n* = 3 for each genotype) in parallel with the viability assay. The Schwann cells were fixed with 4% formaldehyde (Sigma, Cat. #158127) in Dulbecco’s phosphate-buffered saline (DPBS, Sigma, Cat. #D8537) 48 h after stimulation, and immunostained with an antibody against Ki-67. The Ki-67 positive cells and total cells were counted using ImageJ and the percentage of Ki-67 positive cells was calculated.

### Migration Assay

The scratch wound healing assay was used to examine the migration of *Sort1*^–^*^/^*^–^ Schwann cells (*n* = 3 independent experiments). *Sort1*^–^*^/^*^–^ or WT Schwann cells were seeded in an IncuCyte^®^ 96 well ImageLock plate (Satorius, Cat. #4379) at 31,000 cells/cm^3^ (10,000 cells/well) in Schwann cell growth media. The plate was incubated at RT for 20 min followed by incubation at 37°C. When the cells formed a confluent monolayer, the media was changed to starvation media (DMEM containing 2% B27 and 1% heat-inactivated FBS). The cells were starved for 18 h, after which a 700–800 μm scratch was induced using the 96-pin mechanical WoundMaker™ (Satorius, Cat. #4493). The media was replaced with either DMEM containing 2% B27, 1% FBS, 2.5 μM forskolin, and 10 ng/ml neuregulin-1 (negative control), DMEM containing 2% B27, 10% FBS, 2.5 μM forskolin, and 10 ng/ml neuregulin-1 (positive control), or DMEM containing 2% B27, 1% FBS, 2.5 μM forskolin, 10 ng/ml neuregulin-1, and 50 ng/ml of NT-3. The plates were then immediately placed in the IncuCyte Zoom Live-Cell Imaging System from Essen Bioscience and pictures were taken with the 10x objective every 3 h for 48 h. A cell mask was created and adjusted to fit the cell type after which the IncuCyte™ Scratch Wound Cell Migration Software Module (Essen BioScience) was used to calculate the Relative Wound Density (the Relative Wound Density is a measurement of the cell density in the wound area relative to the cell density in the non-wound area).

### Schwann Cell-Dorsal Root Ganglia Neuron Co-cultures

Dorsal root ganglia (DRG) were dissected from neonatal WT rats on postnatal days 1–3 and dissociated with 0.25% trypsin for 30 min at 37°C before being seeded onto poly-L lysine and laminin (Sigma, Cat. #L2020)-coated coverslips. The DRG neurons were purified and maintained in the presence of NGF (100 ng/ml, Promega, Cat. #G514A) for 2 weeks with cycles of maintenance medium (MEM) (Sigma, Cat. #M2279) containing 10% FBS, Primocin (0.1 mg/ml), 0.4% glucose, and GlutaMAX (2 mM, Fisher Scientific, 35050-061), and anti-mitotic medium [DMEM containing Primocin (0.1 mg/ml), pyruvate (1 mM), rat transferrin (10 mg/L, Sigma, Cat. #SRP6513), progesterone (20 nM, Sigma, Cat. #P8783), putrescine (100 μM, Sigma, Cat. #P5780), insulin (5 mg/L, Sigma, Cat. #I6634) supplemented with FdU (10 μM, Sigma, Cat. #F0503), and uridine (10 μM, Sigma, Cat. #U3750)]. Purified *Sort1*^–^*^/^*^–^ or WT Schwann cells were seeded onto the DRG neurons (100.000 cells/well) and allowed to proliferate for 1 week in a maintenance medium before the addition of ascorbic acid (50 μg/ml, Sigma, Cat. #A5960) to induce myelin formation, in the presence or absence of 50 ng/ml NT-3. The medium was changed every 2–3 days. The Schwann cell-DRG neuron co-cultures were fixed for 10 min with 4% formaldehyde in DPBS, 11 days after myelin induction. The cells were permeabilized and unspecific binding of antibodies was blocked with 0.3% Triton-X-100 and 10% FBS in DPBS for 1 h. Subsequently, the cells were incubated with mouse anti-Myelin Basic Protein (1:50, Sigma, Cat. #MAB381) and rabbit anti-neurofilament M (1:200, Millipore, Cat. #AB1987) overnight in a humified box at 4°C. The following day, the Schwann cells were washed 3 times for 10 min in DPBS before 4 h incubation at RT with Alexa Fluor 488 donkey anti-mouse IgG (1:300, Abcam, Cat. #AB150105) and Alexa Fluor 568 donkey anti-rabbit IgG (1:300, Abcam, Cat. #AB175470), diluted in blocking buffer, with Hoechst 33258 (1:10.000, Sigma) as a nuclear stain. The cells were washed and mounted with Fluorescent mounting medium (Dako), sealed with nail polish, and stored at 4°C until image acquisition with the LSM 780 laser scanning confocal microscope (Carl Zeiss) equipped with a Plan-Apochromat 20x/0.8 lens objective (Carl Zeiss). The myelin segments were counted in ImageJ (*n* = 3 for each genotype, 6 images were acquired per coverslip for each experiment, and myelin segments per image averaged).

### Statistical Analysis

Statistical comparison of data was accomplished using one-way or two-way ANOVA or multiple analysis and Bonferroni’s or Bonferroni Holm’s multiple comparisons *post hoc* test, with Graph Pad Prism software. Quantitative data are reported as mean ± SEM. Statistical significance was established for *p < 0.05, **p < 0.01, ***p < 0.001, and ****p < 0.0001.

## Results

### Functional Parameters of Nerve Recovery After Sciatic Crush Injury Are Largely Unaffected in *Sort1*^–^*^/^*^–^ Mice

**FIGURE 1:**
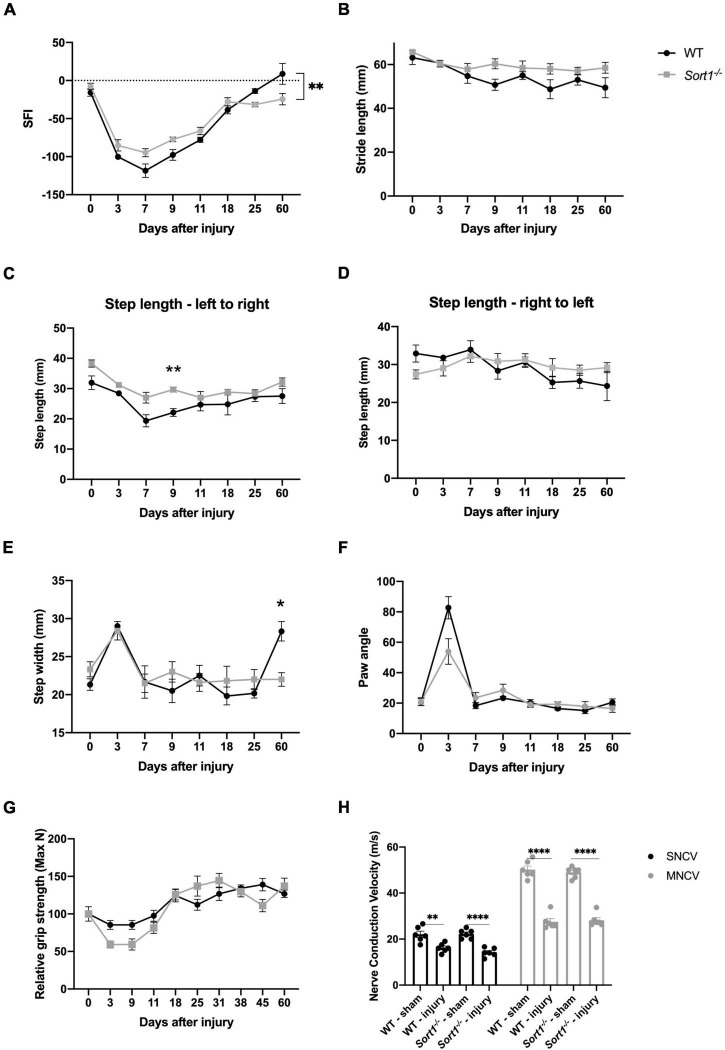
Motor recovery after sciatic nerve crush injury. Motor function recovery of WT and *Sort1*^–^*^/^*^–^ mice assessed by walking tract analysis and calculation of **(A)** SFI, **(B)** stride length, **(C,D)** step length, **(E)** step width, and **(F)** angle of paw placement to the midline, assessed at pre-treatment (time = 0), 3, 7, 9, 11, 18, 25, and 60 days after nerve crush injury. **(G)** Grip strength was additionally assessed at pre-treatment (time = 0), 3, 9, 11, 18, 25, 31, 38, 45, and 60 days after nerve crush injury. **(H)** Nerve conduction velocity analysis of sural (sensory; SNCV) and sciatic (motor; MNCV) nerves 60 days after injury on the ipsilateral and contralateral (sham) side. *N* = 6 WT mice or 6 *Sort1*^–^*^/^*^–^ mice. Statistical significance was established with multiple measures test **(A–G)** or two-way ANOVA **(H)** and Bonferroni’s multiple comparisons *post hoc* test (*p < 0.05, **p < 0.01, ****p < 0.0001).

The sciatic nerve crush model was chosen to investigate the role of sortilin on nerve regeneration. The crush injury model with the intact basal lamina, as opposed to a transection injury where the basal lamina is disrupted, provides a system to test if sortilin enhances or inhibits peripheral nerve regeneration without variables imposed by transection injuries [reviewed in Griffin et al. (2010)]. We have previously described the impact of sortilin on neuropathic pain (Richner et al., 2019), and herein, we focus on functional and morphometric analysis of the injured nerve. The overall functional recovery was evaluated over 60 days by the sciatic functional index (SFI), which is a metric tool to evaluate sciatic nerve function (Inserra et al., 1998). We found that at day 7 post-injury, there was a maximum reduction for WT mice, with a steady recovery over the next few weeks until reaching baseline level from day 25. Although *Sort1*^–^*^/^*^–^ mice displayed a similar overall pattern, the SFI values were significantly reduced on days 25 and 60, relative to both WT and pre-injury baseline (Figure 1A). Next, the recovery was further examined by measuring the dynamic gait parameters (Mendes et al., 2015). Parameters such as stride and step length did not reveal any major altered patterns or endpoints between the genotypes (Figures 1B–D). However, the step width was significantly higher at the endpoint in WT as compared to the Sort1^–/–^ mice (Figure 1E). The paw angle is the angle between the paw axis and the midline of the mouse body during movement and it can be affected by abnormalities of the muscles controlling the paw or the innervating nerves. We correspondingly observed increased paw angle 3 days after injury, which corroborates loss of motor and sensory nerve muscle innervation, but with no significant differences detected between the two genotypes (Figure 1F). Muscle strength was evaluated by the grip strength test at pre-injury baseline (day 0) and at different time intervals up to 60 days after nerve crush, with a recovery pattern for *Sort1*^–^*^/^*^–^ mice indistinguishable from that of WT mice (Figure 1G).

Due to the reduced SFI recovery in *Sort1*^–^*^/^*^–^ mice (Figure 1A), we finally assessed the nerve electrophysiological properties by measuring the motor and sensory nerve conduction velocities (MNCV and SNCV, respectively) at the terminal endpoint day 60. As expected, the injured WT mice displayed a significant reduction in both SNCV and MNCV as compared with pre-injury (Figure 1H). The corresponding parameters for *Sort1*^–^*^/^*^–^ mice were identical to those of WT mice at both naïve or injury conditions (Figure 1H).

### Abnormal Ensheathment of C-Fibers After Crush Injury in *Sort1*^–^*^/^*^–^ Mice

**FIGURE 2:**
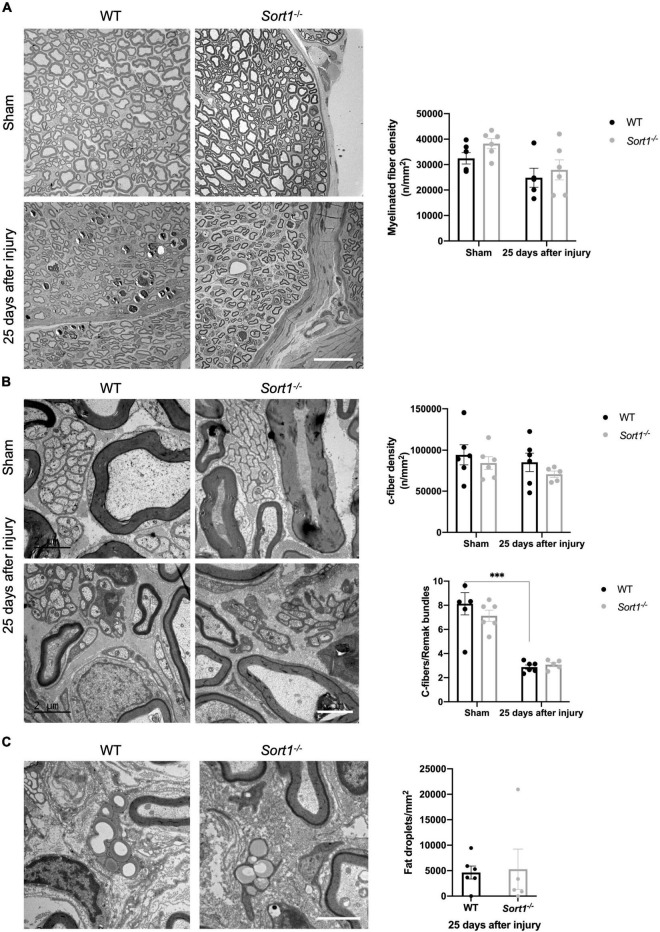
Morphological and morphometrical analysis of crush-injured *Sort1*^–^*^/^*^–^ sciatic nerve. **(A)** Myelinated and **(B)** C-fiber densities were similar between *Sort1*^–^*^/^*^–^ mice and WT mice in the contralateral (sham) and 25 days after sciatic nerve crush injury. Representative EM images of thin transverse sections of sham contralateral or the distal stumps of crush-injured sciatic nerves 25 days after injury [scale bar **(A)**: 20 μm; **(B)**: 2 μm]. Bottom graph in panel **(B)** shows the average number of C-fibers/Remak bundle, which were significantly lower (***p < 0.001) in both crush-injured WT and *Sort1*^–^*^/^*^–^ sciatic nerve compared to the contralateral sham control. **(C)** Representative images of fat droplets in thin transverse sections of injured WT or *Sort1*^–^*^/^*^–^ sciatic nerve 25 days after sciatic nerve crush injury. The total number of fat droplets/mm^2^ were similar between WT and *Sort1*^–^*^/^*^–^.

Histological examination using electron microscopy analysis of the sciatic nerve transverse sections demonstrated overall normal histology of the sciatic nerve in naïve *Sort1*^–^*^/^*^–^ mice (Figure 2). The endoneurium was occupied by myelinated fibers with different caliber sizes (Figure 2A) and unmyelinated axons (C-fibers) that appeared in large groups entailed by Schwann cell cytoplasm, forming Remak bundles (Figure 2B). No difference in myelinated or C-fiber densities was observed between *Sort1*^–^*^/^*^–^ and WT sciatic nerve in naïve conditions (Figures 2A,B), per previous studies (Jansen et al., 2007; Vaegter et al., 2011). As expected, pathology was prominent in the injured tissue of WT mice 25 days after injury. Accordingly, axonal degeneration of myelinated axons and removal or degradation of myelin by Schwann cells was evident. These parameters were indistinguishable between the genotypes (Figures 2A,B), as were the levels of total fat droplets (Figure 2C), indicating that the *Sort1*^–^*^/^*^–^ status did not affect lipid degeneration or reutilization after nerve crush. The densities of myelinated axons (Figure 2A) and C-fibers (Figure 2B), 25 days after injury were also identical between *Sort1*^–^*^/^*^–^ and WT mice.

**FIGURE 3:**
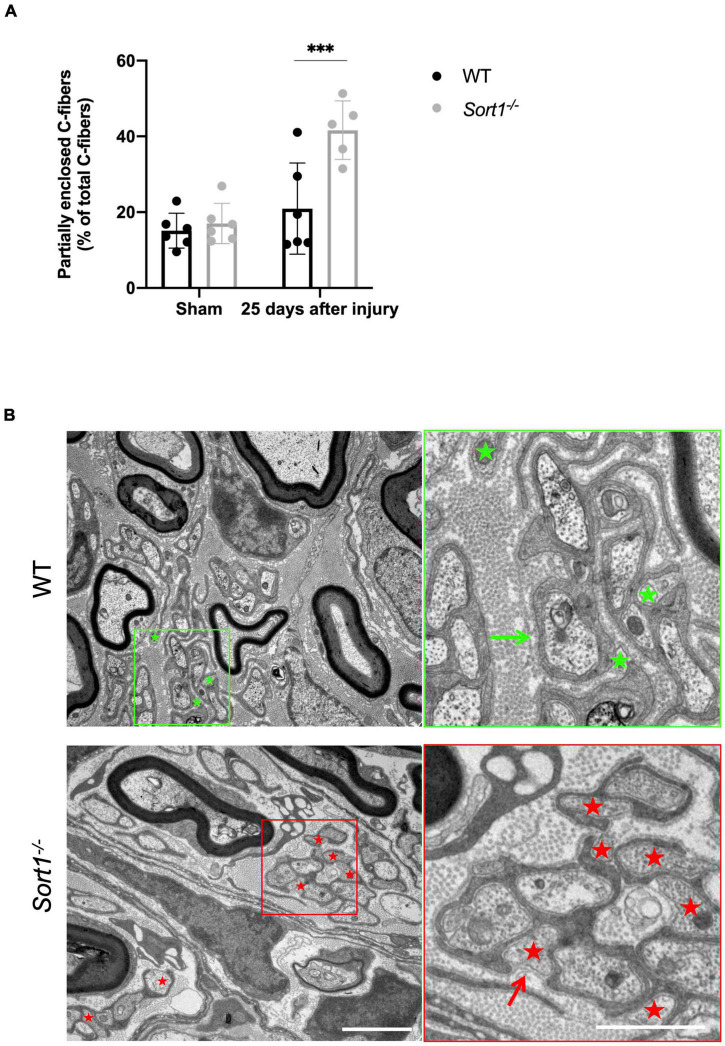
Reduced axon ensheathment by Remak Schwann cells in regenerated *Sort1*^–^*^/^*^–^ sciatic nerve. Significantly more partially enclosed C-fibers were observed in the injured *Sort1*^–^*^/^*^–^ sciatic nerve compared to WT. Representative images showing normal Schwann cell ensheathment of C-fibers in injured WT sciatic nerve 25 days after crush injury (indicated by the green arrow) and only partly enclosure of C-fibers by Schwann cell cytoplasm and partly by only basement membrane 25 days after sciatic nerve crush injury in WT (green stars) and the *Sort1*^–^*^/^*^–^ mouse (indicated by red stars and red arrows). Scale bars: left images 2 μm, right images (zoom of left) 1 μm. **(A,B)** *N* = 6 WT mice and 6 *Sort1*^–^*^/^*^–^ mice. Statistical significance was established with two-way ANOVA and Bonferroni’s multiple comparisons *post hoc* test (***p < 0.001).

For both genotypes, we observed that C-fibers appeared in lower average numbers in the Remak bundles on the injured side as compared to the contralateral sham sciatic nerve (Figure 2B, bottom chart), with extensive Schwann cell cytoplasmic branches spreading throughout the endoneurium. Nonetheless, a prominent feature of the *Sort1*^–^*^/^*^–^ mice entailed a more than twofold increase in the number of C-fibers only partially enclosed by Schwann cell cytoplasm with areas where the C-fiber was naked and not engulfed by the basement membrane (Figures 3A,B), demonstrating a potentially important role for sortilin in the Remak bundle formation following injury.

### Neurotrophin-3 Signaling Is Decreased in *Sort1*^–^*^/^*^–^ Primary Schwann Cells

**FIGURE 4:**
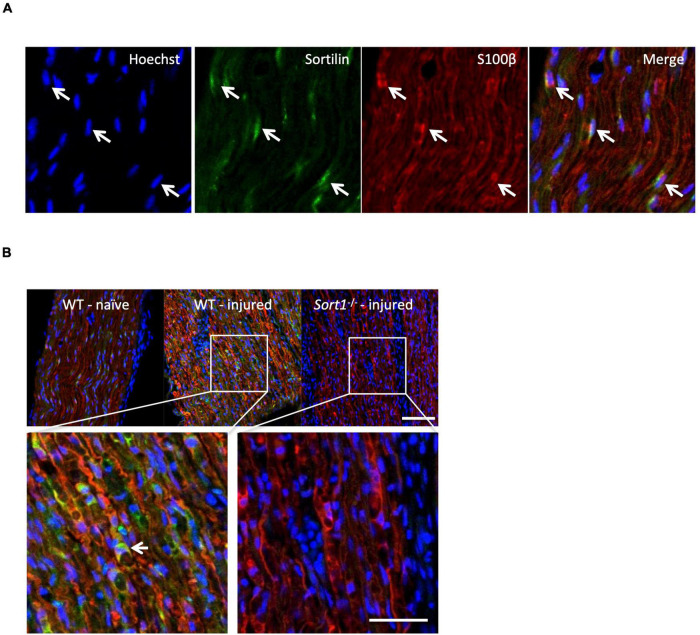
Schwann cells of the sciatic nerve express sortilin. **(A)** Double-labeled immunofluorescence microscopy of sortilin (green) and s100β (red) in longitudinal sections of wild-type (WT) naïve sciatic nerve. Nuclei are labeled with Hoechst (blue). White arrows demonstrate Schwann cells expressing sortilin. Scale bar 50 μm. **(B)** Double-label immunofluorescence microscopy of sortilin (green) and s100β (red; Schwann cell marker) in longitudinal sections of naïve sciatic nerves and 5 days after crush injury in WT and *Sort1*^–^*^/^*^–^ distal sciatic nerves. Nuclei are labeled with Hoechst (blue). The white arrow points to a sortilin-positive (green) s100β-positive (red) WT Schwann cell. Scale bar 100 μm upper images and 50 μm lower images.

**FIGURE 5:**
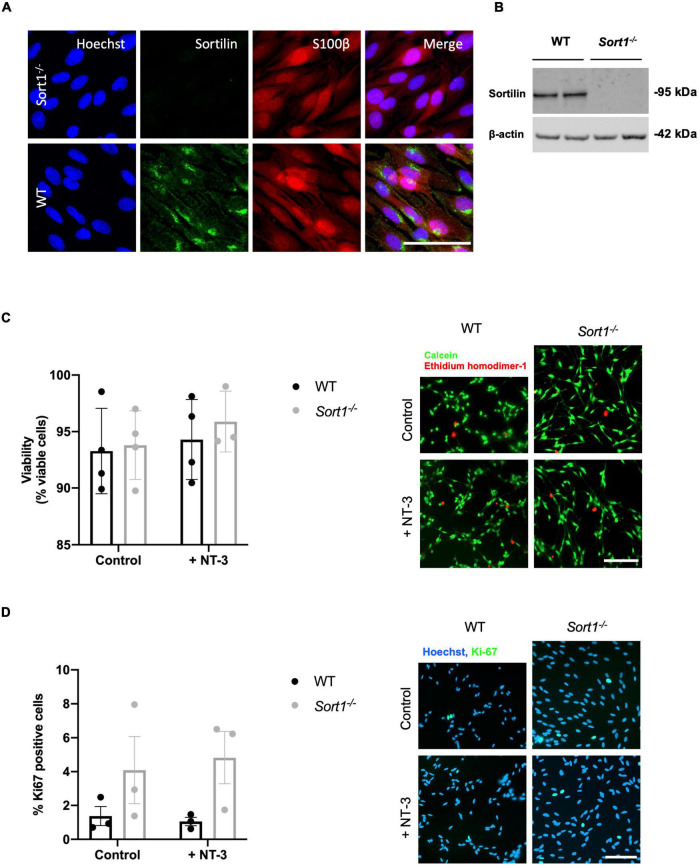
Primary cultured Schwann cells express sortilin. **(A)** Double-labeled immunofluorescence microscopy of primary cultured Schwann cells show sortilin (green) in wild-type (WT) Schwann cells (red; identified by the Schwann cell marker s100β), but not in cells derived from *Sort1*^–^*^/^*^–^ rat sciatic nerves. Nuclei are labeled with Hoechst (blue). Scale bar 50 μm. **(B)** Immunoblot analysis of sortilin in lysates of WT or *Sort1*^–^*^/^*^–^ primary Schwann cells shows sortilin expression in WT. **(C)** The viability of *Sort1*^–^*^/^*^–^ Schwann cells is like WT Schwann cells with (95.89 ± 1.55% vs. 94.30 ± 1.77%, *p* > 0.99) or without NT-3 stimulation (93.8 ± 1.52% vs. 93.29 ± 1.89%, *p* > 0.99). Viable cells were identified in Schwann cell cultures 24 h after stimulation with or without NT-3 by calcein (green) fluorescence, while dead cells were identified by ethidium homodimer-1 (red) fluorescence. Scale bar 100 μm. Viability reflects mean% calcein-positive cells ± SEM, *n* = 4. **(D)** Schwann cell proliferation (percentage of Ki-67% cells) was not significantly different in cultures of *Sort1*^–^*^/^*^–^ Schwann cells compared to WT Schwann cell cultures with (4.82 ± 1.54% vs. 1.05 ± 0.25%, *p* = 0.4381) or without NT-3 stimulation (4.09 ± 1.98% vs. 1.37 ± 0.56%, *p* > 0.99). The proliferation marker, Ki-67 (green), was identified by immunofluorescence in Schwann cell cultures 48 h after stimulation with or without NT-3. Nuclei are labeled with Hoechst (blue). Scale bar 100 μm. Proliferation rates reflect% Ki-67 positive cells ± S.E.M., *n* = 3. Statistical significance was analyzed using two-way ANOVA with Bonferroni’s multiple comparisons *post hoc* test.

The sortilin expression was confirmed by immunohistochemistry in Schwann cells of naïve WT murine sciatic nerves co-localizing with the Schwann cell marker S100β (Figure 4A). Furthermore, the sciatic crush injury resulted in considerably increased sortilin immunoreactivity in Schwann cells at 5 days post-injury (Figure 4B). These *in vivo* observations prompted us to investigate the potential involvement of sortilin on Schwann cell neurotrophin signaling using primary cell cultures. Immunofluorescence (Figure 5A) and WB analysis (Figure 5B) confirmed sortilin expression in cultured WT Schwann cells with no detectable sortilin in *Sort1*^–^*^/^*^–^ Schwann cells. Quantification of viability and proliferation, as addressed by the live and death assay (Figure 5C) and the Ki67-positive cell percentages (Figure 5D), respectively, were indistinguishable between WT and *Sort1*^–^*^/^*^–^ Schwann cells, indicating that *Sort1* expression is not essential for Schwann cell proliferation or viability.

**FIGURE 6:**
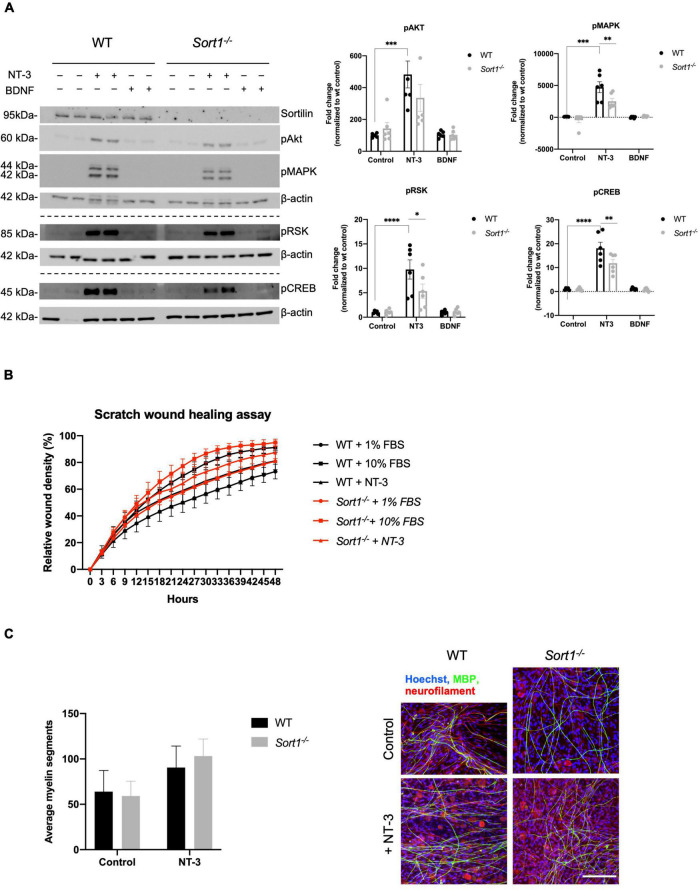
Neurotrophin signaling is decreased in *Sort1*^–^*^/^*^–^ Schwann cells. **(A)** pAkt, pMAPK, pRSK, and pCREB were quantified by densitometry of immunoblots from lysates of NT-3 or BDNF stimulated wild-type (WT) or *Sort1*^–^*^/^*^–^ primary cultures of Schwann cells and normalized against β-actin. pMAPK, pRSK, and pCREB levels are significantly lower in *Sort1*^–^*^/^*^–^ Schwann cells compared to WT Schwann cells. Data represent the mean ± SEM for *n* = 3. **(B)** The relative wound healing closure time was similar in *Sort1*^–^*^/^*^–^ Schwann cells relative to WT Schwann cells with or without 10% FBS (positive control) or NT-3. Furthermore, the relative wound healing closure time indicates that NT-3 does not increase the relative wound healing closure time significantly in WT Schwann cells compared to unstimulated controls; 10% FBS significantly increased the relative wound healing closure time at 27–39 h (*p < 0.05). **(C)** *Sort1*^–^*^/^*^–^ Schwann cells showed similar myelin formation relative to WT Schwann cells. Co-cultures of DRG neurons and Schwann cells were fixed 11 days after myelin induction with or without NT-3 stimulation. The myelin segments were identified with an antibody against myelin basic protein (MBP; green) and the neurites were identified with antibodies against neurofilament (red). Hoechst identifies nuclei (blue). Statistical significance was determined by two-way ANOVA with Bonferroni’s multiple comparisons *post hoc* test. Data represents mean ± SEM, *n* = 3 (*p < 0.05, **p < 0.005, ***p < 0.001, ****p < 0.0001).

We have previously demonstrated the involvement of sortilin in neurotrophin signaling in dorsal root ganglia neurons (Vaegter et al., 2011). To further examine such a role in Schwann cells, primary WT and *Sort1*^–^*^/^*^–^ cultures were stimulated with either BDNF or NT-3. Using WB analysis, we found that NT-3 stimulation significantly increased the pAKT, pMAPK/ERK, pRSK, and pCREB protein levels in WT Schwann cells (Figure 6A). In contrast, BDNF did not activate these pathways, confirming previous reports that Schwann cells do not express the full-length catalytically active form of the BDNF neurotrophin receptor TrkB (Funakoshi et al., 1993). Interestingly, relative to WT, NT-3 stimulation of *Sort1*^–^*^/^*^–^ Schwann cells significantly reduced the pMAPK/ERK response by 47 ± 10%, while the downstream mediators, pRSK and pCREB, were significantly reduced by 45 ± 15 and 35 ± 9%, respectively (Figure 6A). As NT-3 has been reported to facilitate Schwann cell migration (Yamauchi et al., 2003, 2005a), we compared WT and *Sort1*^–^*^/^*^–^ Schwann cell migration in the scratch wound healing assay but observed similar migration rates between the two groups of primary Schwann cells (Figure 6B). NT-3 is furthermore described to inhibit myelination (Chan et al., 2001) and we compared the performance of WT and *Sort1*^–^*^/^*^–^ Schwann cells in their ability to myelinate segments of WT dorsal root ganglia neurons in an *in vitro* co-culture system. While we obtained a similar degree of myelination for both genotypes in the absence of NT-3, we were also unable to confirm an inhibitory effect of NT-3 on myelination in either genotype but rather observed a (non-significant) trend toward a stimulatory effect (Figure 6C).

In conclusion, sortilin ablation in cultured primary Schwann cells reduced the activation of NT-3 stimulated intracellular signaling pathways. Surprisingly, we did not observe any NT-3-dependent cellular responses in either genotype on *in vitro* characteristics of proliferation, survival, migration, or myelination.

## Discussion

Sortilin facilitates neurotrophin signaling in DRG neurons by anterograde trafficking of Trk receptors and even though sortilin ablation does not seem to affect the development of the PNS (Jansen et al., 2007; Vaegter et al., 2011), a role of sortilin in nerve regeneration following, e.g., a crush injury, has not been studied. Here, we demonstrate that while nerve regeneration appears to be functionally normal following sciatic nerve crush in *Sort1*^–^*^/^*^–^ mice, neuroanatomical examination revealed abnormal structural regeneration demonstrated by the impaired embracing of C-fibers by the Remak Schwann cells. *In vitro* studies further demonstrated a decreased signaling response to NT-3 in primary cultures of *Sort1*^–^*^/^*^–^ Schwann cells, with blunted activation (phosphorylation) of MAPK/ERK, RSK, and CREB. Naïve *Sort1*^–^*^/^*^–^ mice display a normal response to acute mechanical and thermal stimuli (Vaegter et al., 2011), but the sensory phenotype of *Sort1*^–^*^/^*^–^ mice is affected by changes in spinal plasticity (Richner et al., 2019). Consequently, a further investigation of a peripheral sensory recovery after nerve crush would likely be concealed by the spinal phenotype of *Sort1*^–^*^/^*^–^ mice and was therefore not addressed in this study.

Schwann cells are remarkably plastic cells, with their Remak or myelin phenotypes highly depending on signals derived from the cellular environment. Furthermore, upon nerve injury and the conversion to a dedifferentiated/repaired Schwann cell type, it seems that the subsequent redifferentiation to a Remak or myelinating phenotype is independent of their prior identity. The MAPK/ERK pathway has a central role in these processes being required for processes in Wallerian degeneration and the subsequent Schwann cell differentiation and remyelination (Harrisingh et al., 2004; Napoli et al., 2012). Different levels or duration of MAPK/ERK activity may, however, elicit distinct responses. An example of this is a study by Cervellini et al. (2017), utilizing a mouse model with sustained MAPK/ERK activation in adult Schwann cells. Following crush injury, post-injury myelin clearance was accelerated while a delay in repair and functional recovery was observed. At 4 weeks following injury, myelinated axons in the transgenic mice demonstrated higher rates of myelin decompaction, a reduced number of Cajal bands, and decreased internodal length. Furthermore, the authors reported the presence of abnormal Remak bundle formations and a reduced number of C-fibers per Remak bundle in the regenerated nerve (Cervellini et al., 2017).

The neurotrophins NGF, BDNF, NT-3, and NT-4 are potent activators of the MAPK/ERK pathway *via* their cognate Trk tyrosine kinase receptors, and their effects on PNS development and signaling have been extensively studied (Chao, 2003; Reichardt, 2006). Schwann cells express TrkC, allowing for NT-3-mediated activation of downstream MAKP/ERK and AKT signaling pathways. They further express a truncated form of TrkB lacking the intracellular tyrosine kinase domain, rendering BDNF/TrkB unable to activate these same pathways. However, BDNF appears to induce signaling in these cells *via* the common neurotrophin receptor p75^NTR^, activating downstream pathways distinct from those of TrkB (Cosgaya et al., 2002; Skeldal et al., 2011). *In vitro* studies have described how NT-3 enhances Schwann cell migration and proliferation (Yamauchi et al., 2003, 2004, 2005a,b), while inhibiting myelin formation in co-cultures of DRG neurons and Schwann cells (Chan et al., 2001; Ng et al., 2007). This is consistent with other reports of the MAPK/ERK pathway involvement in these events. Accordingly, stimulation of the MAPK/ERK pathways facilitated *in vitro* Schwann cell migration while application of MAPK/ERK inhibitor impaired migration (Meintanis et al., 2001). Inhibition of this pathway was also found to block Schwann cell proliferation *via* inhibition of cyclin/cdk activity (Lloyd et al., 1997). Intriguingly, while we observed robust MAPK/ERK activation in NT-3 stimulated cultures, we were unable to detect any effect on WT Schwann cell migration as well as on their rate of proliferation. However, our data seem consistent with a study by Woolley et al. (2008) examining NT-3-deficient mice. These authors found that ablation of NT-3 expression did not affect developmental myelination in terms of timing or structural features. Furthermore, from postnatal day 21 when myelination was complete, a reduction in MAG and P0 expression levels and myelin thickness were observed (Woolley et al., 2008), seemingly contradicting predictions based on *in vitro* studies that removal of an inhibitory NT-3 signal would have been expected to enhance myelination. Further support for a myelin-promoting role of NT-3 came from a study on NT-3 heterozygote mice subjected to a nerve crush injury, where reduced NT-3 expression was found to decrease myelination of regenerating axons (Sahenk et al., 2008). Graft implantation studies indicated that Schwann cell-secreted NT-3 is necessary for post-injury remyelination, apparently acting *via* an autocrine-signaling loop (Meier et al., 1999; Sahenk et al., 2008). In terms of Remak Schwann cells, NT-3/TrkC also seems to play a substantial role. Accordingly, it was found that the administration of activating anti-TrkC antibodies to a nerve crush model in trembler mice increased the presence of Remak fibers, as well as increased functional recovery measured by grip strength but with no impact on NCV (Sahenk et al., 2010). In another injury study, acellular nerve grafts populated with Schwann cells genetically modified to express NT-3 resulted in the presence of twice as many Remak bundles compared to grafts populated with WT Schwann cell controls, although a reduction in the number of axons per bundle was also found (Godinho et al., 2012). These latter studies point toward a stimulatory role of NT-3/TrkC on the regeneration of Remak bundles, which is consistent with our data presented here that loss of sortilin blunts NT-3 signaling in Schwann cells and impairs Remak bundle regeneration. Interestingly, we have previously described an effect of sortilin on the structural integrity of Remak bundles. Combined deletion of sortilin and p75^NTR^ (*Sort1^–/–^; Ngfr^–/–^*) displayed normal Remak bundle development at postnatal day 15 but later these structures underwent a marked degeneration. The mice with deletion of either receptor alone did not present any spontaneous Remak degenerative phenotype (Vaegter et al., 2011). It, therefore, seems that neurotrophin signaling in the PNS is governed by a rather robust system with significant buffer capacity as the system can be pressured without developmental consequences. This is further illustrated by the normal structural development of peripheral nerves in the mice heterozygous for TrkA, -B or -C (Klein et al., 1993; Ernfors et al., 1994b,a; Minichiello et al., 1995), or p75^NTR^ (Lee et al., 1992). The biological role of sortilin in neurotrophin signaling needs to be unmasked in situations where the system is closer to its limit, as exemplified here by nerve crush or in our previous study by p75^NTR^ deletion (Vaegter et al., 2011), both affecting the Remak bundle structure. The latter study further identified severe sympathetic neuropathy upon sortilin deletion in the TrkA heterozygous mice.

NT-3/TrkC stimulation as well as the temporal activation and intensity of MAPK/ERK activation seems essential to facilitate the regenerative process in terms of remyelination and formation of Remak bundles. Here, we describe how sortilin immunoreactivity is increased in the nerve shortly after injury and how sortilin ablation results in Remak pathology. The observation of blunted NT-3 signaling response in cultured *Sort1^–/–^* Schwann cells supports a function of sortilin in regulating NT-3 signaling in Remak Schwann cells following nerve injury.

## Data Availability Statement

The raw data supporting the conclusions of this article will be made available by the authors, without undue reservation.

## Ethics Statement

The animal study was reviewed and approved by Danish Animal Experiments Inspectorate under the Ministry of Justice (permission no. 2017-15-0201-01192).

## Author Contributions

MU, NG, and CV designed the experiments and wrote the manuscript. MU, NG, SMoh, SH, TL, and NM performed the experiments. MU, NG, SMoh, SH, SMol, OA, ÅS, SG, AN, and CV interpreted the results, and contributed with reagents, materials, and analysis tools. All authors contributed to the article and approved the submitted version.

## Conflict of Interest

The authors declare that the research was conducted in the absence of any commercial or financial relationships that could be construed as a potential conflict of interest.

## Publisher’s Note

All claims expressed in this article are solely those of the authors and do not necessarily represent those of their affiliated organizations, or those of the publisher, the editors and the reviewers. Any product that may be evaluated in this article, or claim that may be made by its manufacturer, is not guaranteed or endorsed by the publisher.
